# A Need for More Molecular Profiling in Brain Metastases

**DOI:** 10.3389/fonc.2021.785064

**Published:** 2022-01-25

**Authors:** Erica Shen, Amanda E. D. Van Swearingen, Meghan J. Price, Ketan Bulsara, Roeland G. W. Verhaak, César Baëta, Brice D. Painter, Zachary J. Reitman, April K. S. Salama, Jeffrey M. Clarke, Carey K. Anders, Peter E. Fecci, C. Rory Goodwin, Kyle M. Walsh

**Affiliations:** ^1^ Division of Neurosurgery, Department of Surgery, University of Connecticut, Farmington, CT, United States; ^2^ The Jackson Laboratory for Genomic Medicine, Farmington, CT, United States; ^3^ Division of Medical Oncology, Department of Medicine, Duke University Medical Center, Durham, NC, United States; ^4^ Duke Center for Brain and Spine Metastasis, Duke Cancer Institute, Duke University Medical Center, Durham, NC, United States; ^5^ Department of Neurosurgery, Duke University Medical Center, Durham, NC, United States; ^6^ Department of Neurosurgery, Cancer Center Amsterdam, Amsterdam Universitair Medische Centra (UMC), Vrije Universiteit Amsterdam (VU) University Medical Center (VUmc), Amsterdam, Netherlands; ^7^ Department of Radiation Oncology, Duke University Medical Center, Durham, NC, United States

**Keywords:** brain metastases, sequencing, biomarkers, neurosurgery, discordance

## Abstract

As local disease control improves, the public health impact of brain metastases (BrM) continues to grow. Molecular features are frequently different between primary and metastatic tumors as a result of clonal evolution during neoplasm migration, selective pressures imposed by systemic treatments, and differences in the local microenvironment. However, biomarker information in BrM is not routinely obtained despite emerging evidence of its clinical value. We review evidence of discordance in clinically actionable biomarkers between primary tumors, extracranial metastases, and BrM. Although BrM biopsy/resection imposes clinical risks, these risks must be weighed against the potential benefits of assessing biomarkers in BrM. First, new treatment targets unique to a patient’s BrM may be identified. Second, as BrM may occur late in a patient’s disease course, resistance to initial targeted therapies and/or loss of previously identified biomarkers can occur by the time of occult BrM, rendering initial and other targeted therapies ineffective. Thus, current biomarker data can inform real-time treatment options. Third, biomarker information in BrM may provide useful prognostic information for patients. Appreciating the importance of biomarker analyses in BrM tissue, including how it may identify specific drivers of BrM, is critical for the development of more effective treatment strategies to improve outcomes for this growing patient population.

## Highlights

- The genomic status of BrM can alter treatment plans for patients by providing new targetable options.
- Molecular profiling of BrM can indicate that a therapy is no longer effective for a patient.
- Biomarker information in BrM may provide useful prognostic information for patients.

## Introduction

Far exceeding primary central nervous system (CNS) neoplasms in number, metastases to the brain pose a significant societal burden. Of an estimated 1.7 million new cancer diagnoses per year in the United States, approximately 6%–14% of these patients are expected to ultimately develop a metastasis to the brain (1–3). Brain metastases (BrM) most commonly arise in patients with primary lung, breast, and melanoma neoplasms but are also observed in patients with renal cell carcinoma, prostate cancer, colorectal cancer, and many other primary cancer histologies (4).

Patients with BrM face a dismal prognosis, with a median overall survival of <6 months regardless of primary cancer type based on historical data (5, 6). Clinically actionable molecular biomarkers, such as genetic alterations and aberrant gene expression, have been increasingly identified and translated into treatment options for cancer patients, with more specific emphasis placed on patients with BrM in recent years (7). Identifying accurate molecular biomarkers for BrM is crucial to developing more effective therapies and advancing personalized oncology care.

Modern management of BrM involves multidisciplinary consideration of surgery, radiation therapy, and systemic therapy options. Surgical resection of BrM provides a survival advantage for patients with a single metastasis (8). In modern practice, neurosurgical resection is considered for patients with a limited number of BrM, for larger metastases, for metastases that can be safely resected, when tissue is needed for diagnosis, and when debulking is needed to alleviate symptoms. Historically, patients were treated with whole-brain radiation therapy (WBRT) either alone or after surgical management given the ability for WBRT to extend intracranial progression-free survival (9). However, modern radiation treatments have shifted toward approaches that seek to mitigate the neurocognitive side effects of WBRT, such as hippocampal avoidant WBRT with memantine (10) or stereotactic radiosurgery (SRS) directed only at the BrM without WBRT. This is due to the ability for SRS to mitigate the neurocognitive side effects of WBRT, while providing comparable overall survival and local intracranial control outcomes (albeit at a cost of decreased distant intracranial control) (11). Increasingly, systemic therapies including chemotherapy, targeted therapies, and immunotherapies are applied for BrM patients. The identification of select BrM patients for whom surgery or radiotherapy can be deferred while the patients are treated with systemic therapies is a topic of investigation for many cancer subtypes.

When surgical management is a primary BrM treatment strategy, biomarker analyses of BrM tissues can offer additional clinical gains. Surgical intervention is often indicated for BrM that are >3 cm, situated in an accessible and/or superficial location, or causing mass effect on the brain (12). Currently, obtaining a tissue biopsy for the primary or sole indication of assessing biomarker information in BrM is not routinely performed due to associated clinical risks in a patient population with a relatively poor prognosis. Biopsies of BrM, including concurrent biopsies obtained during laser interstitial thermal therapy (LITT) (13), are routinely sent to pathology for diagnostic confirmation and/or differentiation from radiation necrosis. However, these biopsies are rarely sent for broad molecular profiling despite an overall increase in the use of commercial and in-house genomic and transcriptomic sequencing services as part of routine oncology care (14, 15).

It has been generally accepted that cancer progression involves somatic clonal evolution (16). Biomarkers identified from primary tumor resections are often assessed years prior to development of BrM and may not reflect emergence of resistance mechanisms that arise during the metastatic process and under treatment pressures. Molecular biomarkers presenting in distant metastases are frequently different from those initially presenting in primary sites. Studies demonstrate that biopsies of other extracranial metastatic sites also do not fully recapitulate the molecular features of BrM—due in part to clonal evolution during neoplasm migration and systemic treatment (16–19). Comparisons of the somatic landscape across visceral metastases may fail to take into account the unique requirements for BrM, such as enabling extravasation through non-fenestrated capillaries, hypoxia-induced neoangiogenesis, and adaptation to the CNS metabolic microenvironment (20). Newer and less invasive techniques for biomarker testing have emerged in recent years (e.g., liquid biopsies). In a recent study, next-generation sequencing of cell-free DNA (cfDNA) from cerebrospinal fluid was shown to be more sensitive than cytologic analysis for diagnosing leptomeningeal disease (21). In the future, cfDNA may be a beneficial tool to detect potential actionable biomarkers in BrM. The use of liquid biopsies to evaluate the response of metastatic tumors to treatment and to provide prognostic information still warrants future investigation (22).

Here, we review the discordance of clinically actionable biomarkers measured in BrM from lung cancer, breast cancer, and melanoma compared to primary sites and extracranial metastases. We discuss these emerging data within the framework of three principal motivations for increased molecular profiling in BrM. First, new treatment targets may be identified as unique actionable mutations emerge in BrM compared to the primary tumor or extracranial metastases. Second, BrM molecular profiling may identify biomarkers of resistance or loss of actionable alterations, thereby excluding ineffective therapies from a treatment plan. Third, new biomarker information in BrM could provide useful prognostic information to aid clinicians and patients in discussing expectations for care.

Obtaining genomic sequencing data on BrM will also help to identify novel drivers that may play a key role in promoting BrM. In a recent report where the authors performed whole-exome sequencing of brain metastases from lung adenocarcinomas (BM-LUAD) and primary lung adenocarcinomas using case–control analysis to identify genomic alterations that promote BrM, they identified three regions (*MYC*, *YAP1*, *MMP13*) that had significantly higher amplification frequencies and one region (*CDKN2A/B*) that had higher deletion frequencies in BM-LUAD as compared to primary lung adenocarcinoma (23). Additional investigations will be needed to identify driver somatic alterations that promote brain metastases in other types of primary tumors.

While some clinicians may be daunted by the variety and complexity of biomarker testing options available, the impact of this hurdle is rapidly diminishing as “omics” data are increasingly incorporated into oncology practice. However, comprehensive molecular profiling of BrM tissues remains an underutilized option in most health systems, especially outside of dedicated multidisciplinary BrM services. The development of a common understanding among healthcare professionals of the importance of biomarker analysis in BrM will be critical for the development of more effective treatment strategies against BrM and the advancement of precision oncology approaches in this growing patient population.

## Identification of New, Actionable Targets

BrM tissue, obtained through either biopsies or surgical resection during standard care, can provide additional opportunities to identify new targets that were not present in primary tumors and that diverge from paired extracranial metastases. In seminal work on the molecular divergence of BrM, Brastianos et al. observed that more than half of BrM studied harbored at least one potentially actionable biomarker that was not present in the paired primary neoplasm (24). Their data from lung, breast, and renal cell cancers further demonstrated that these alterations were often unique to BrM when compared to lymph node and other extracranial metastases (24). These results have been supported by other recent analyses identifying potentially new and actionable biomarkers in BrM arising from non-small cell lung cancer (NSCLC), breast cancer, and melanoma, described below and summarized in **Table 1**.

**Table 1:** Summary of therapeutic possibilities and prognostic information associated with biomarkers in brain metastases.

	Biomarkers (types)	Mechanisms of Actions	Discordance Rates Between BrM and Primary And Extracranial Neoplasm Sites	Therapeutic Options if Biomarkers Are Present In BrM	Alternative Therapeutic Options if Drug Resistance Has Occurred	Associated Prognostic Information
**NSCLC**	EGFR (mutation)	Receptor tyrosine kinase	19%–66.7% (25, 26)	TKIs: afatinib; erlotinib or gefitinib + radiotherapy or chemotherapy (27–29)	Osimertinib targeting EGFR T790M (30, 31)	↑ PFS in EGFR-mutant tumors treated with icotinib vs. uncommon EGFR mutations (32)
	ALK (rearrangement)	Receptor tyrosine kinase	ALK fusion: rare	TKIs: ceritinib, alectinib, brigatinib, or lorlatinib (34–39)		
			ALK amplification w/o fusion: 12.5% (33)			
	ROS1 (rearrangement)	Receptor tyrosine kinase	ROS1 fusions enriched in BrM (26)	TKIs: entrectinib, lorlatinib, ceritinib (40, 41)		
	MET (mutation/overexpression)	Receptor tyrosine kinase	Mutations and amplifications enriched in BrM (42)	TKIs: tepotinib, capmatinib (43–45)	Possibly contributing to EGFR treatment resistance; combination therapies under investigation (42, 46–50)	
	RET (mutation/rearrangement)	Receptor tyrosine kinase		TKIs: selpercatinib, pralsetinib (51–54)		
	KRAS (overexpression/mutation)	GTPase	13% (55)	TKIs: sotorasib (G12C) (56)		
**Breast cancer**	ER/PR (expression/mutation)	Hormone receptor	ER: 13.6%–29.2% (57–59)	Endocrine therapy: tamoxifen (57, 58)		
			PR: 4.2%–44.4%			
	HER2 (overexpression/mutation)	Receptor tyrosine kinase	2.3%–23.8% (57–60)	Anti-HER2: trastuzumab, pertuzumab, lapatinib (14, 61)		↑ OS likely attributed to treatment effects (59)
				anti-AR: bicalutamide or enzalutamide (62, 63)		
	PTEN (loss)	Regulation of PI3K/AKT/mTOR pathway	Loss of PTEN is often seen in BrM, but is less commonly seen in extracranial sites (64–66)	PARP inhibitors: olaparib, veliparib (7, 67, 68)	Single-targeting therapies often found ineffective; combination therapies currently under investigation (e.g., HER3+PI3K or PI3K+mTOR) (69, 70)	↓ time to tumor recurrence in a distant site (63)
						↓ OS in TNBC subtypes (71, 72)
	CDK pathway (mutation/loss)	Serine/threonine protein kinase; regulation of G1 checkpoint	Clinically actionable alterations in the CDK pathway genes in 28% of BrM not seen in primaries (24)	CDK4/6 inhibitors: abemaciclib, palbociclib, ribociclib (24, 73, 74)		
	RB1 (loss)	Regulation of G1 checkpoint	RB1 loss more commonly observed in BrM (24)		May contribute to CDK4/6 inhibitor treatment resistance (24, 73, 74)	
	HK2 (overexpression)	Glucose metabolism				↓ post-craniotomy survival in breast cancer patients w/BrM (75)
**Melanoma**	BRAF (mutation)	Serine–threonine kinase	7% (76, 77)	TKIs: vemurafenib, dabrafenib (78)		

RTKis, receptor tyrosine kinase inhibitors; OS, overall survival; PFS, progression-free survival.

### Non-Small Cell Lung Cancer

Among the various biomarkers associated with lung cancer, genetic alterations in *epidermal growth factor receptor* (*EGFR*) are perhaps the most notable biomarker affecting the management of NSCLC patients with BrM. Previous reports have observed a discordance rate of *EGFR* mutation status between paired BrM and corresponding primary lung tumor samples from 19% to as high as 67% (25, 26), with BrM typically displaying a higher frequency of *EGFR* mutations than primary NSCLC tumors (79). Identification of *EGFR* mutations in BrM presents treatment opportunities, as studies suggest that first-generation EGFR tyrosine kinase inhibitors (TKIs), such as gefitinib and erlotinib, have CNS activity (80–83). It is important to note that patients with NSCLC BrM who received erlotinib or gefitinib plus radiotherapy or chemotherapy have exhibited significant intracranial responses and experienced longer progression-free survival (PFS) and median overall survival (OS) compared with patients who received erlotinib or gefitinib alone (27, 28). Similar considerations can be entertained for patients receiving the third-generation EGFR TKI osimertinib, which has emerged as an attractive first-line treatment for NSCLC and for NSCLC harboring *EGFR* Thr790Met (T790M) mutations (29).

Anaplastic lymphoma kinase (ALK) is another notable biomarker in the management of NSCLC patients with BrM (84, 85). The most prevalent *ALK* alteration involves the fusion of *ALK* with the *microtubule-associated protein-like 4 gene (EML4)*. The fusion event results in the autophosphorylation and constitutive activation of ALK kinase, which contributes to tumorigenesis and progression (86, 87). Current data suggest that the concordance for *ALK* gene fusion between the primary neoplasm site and the matched BrM appears high (33). Knowing the *ALK* mutation status in BrM is critical, as several drugs exhibiting CNS penetrance, in particular alectinib, brigatinib, and lorlatinib, have been approved by the FDA for the treatment of *ALK*-fusion-positive metastatic NSCLC (34, 35). Alectinib, brigatinib, and lorlatinib have all been demonstrated in clinical trials (Alex, ALTA-1L, and Crown) to have superior efficacy to crizotinib in the primary treatment of *ALK*-positive NSCLC (36–38). Intracranial response rates in these and other trials indicate that brigatinib and lorlatinib have significant efficacy against *ALK*-positive BrM (38, 39, 88), although the effectiveness of these agents on *ALK*-amplified BrM requires further investigation.

Many biomarkers demonstrate a significant rate of concordance between primary tumor sites and BrM. Nevertheless, routine molecular profiling of BrM will help identify possible new actionable biomarkers, especially when there are approved therapeutic options that exhibit good blood–brain barrier permeability, which were recently elegantly reviewed by Soffietti and colleagues (7). These targets include *ROS Proto-Oncogene 1 (ROS1)*, *MET Proto-Oncogene* (*MET) exon 14 skipping mutation*, *RET Proto-Oncogene* (*RET)*, *Neurotrophic Receptor Tyrosine Kinase* (*NTRK)*, *B-Raf Proto-Oncogene* (*BRAF)*, and *KRAS Proto-Oncogene* (*KRAS).* Both crizotinib and entrectinib, multi-targeted TKIs, are now U.S. Food and Drug Administration (FDA)-approved for treatment of NSCLC patients with *ROS1*-rearranged mutations (40, 43). However, as noted above, crizotinib has demonstrated limited intracranial efficacy in the clinic, while studies with entrectinib have reported intracranial response rates of up to 55% (89). Studies with lorlatinib and ceritinib in *ROS1*-positive NSCLC have also demonstrated high rates of intracranial response in patients with BrM (41, 88). In a Phase 2, open-label study, approximately 50% of NSCLC patients with *MET* exon 14 skipping mutations had some response to treatment with tepotinib and capmatinib (43, 44). Both capmatinib and tepotinib are FDA-approved for treatment of patients with *MET* exon 14 skipping mutant metastatic NSCLC, and recent studies report promising intracranial responses to both agents in patients with this mutation (44, 45). Selpercatinib and pralsetinib, two highly selective inhibitors of RET kinase, have been recently approved by the FDA for use in NSCLC patients with *RET* mutations (51, 52) and have both shown robust intracranial activity in patients with BrM (53, 54). *KRAS* is frequently altered in NSCLC, either through activating mutations or through amplification (55). The FDA approved in 2021 the first KRAS inhibitor, sotorasib, which specifically targets the G12C mutant form of *KRAS*, for metastatic NSCLC. Recent work in matched lung adenocarcinoma primary and BrM tissues reported that KRAS alterations were present in 13% of BrM tissues that were not present in the matched primary, with enrichment of G12C and G13C mutations (55). Given the potential intracranial efficacy of sotorasib (56) and ongoing trials to address this question, identification of a *KRAS* G12C mutation in a BrM may provide a potential new avenue for directed therapy in these patients.

### Breast Cancer

Among a host of biomarkers important for the clinical management of breast cancer (BC), estrogen receptor (ER), progesterone receptor (PR), and HER2 are the most crucial. Hormone receptor (HR; ER or PR)-negative, HER2-positive, and triple-negative (TNBC; ER-, PR-, HER2-) statuses are associated with increased risk for BCBrM (90). High discordance in these biomarkers exists between primary BC and BrM: ER: 13.6%–29.2%, PR: 4.2%–44.4%, and HER2: 2.3%–23.8% (57–59). In a recent large analysis, this discordance led to subtype switching between primary tumors and BrM in 22.8% of patients (91, 92). Furthermore, pathology and mRNA expression analyses have revealed a downregulation of ER (*ESR1*) and PR (*PGR*) gene expression and an upregulation of HER2 (*ERBB2*) gene expression in BrM, particularly in those arising from TNBC (19, 60, 91, 92).

Since HR and HER2 status are frequently used to determine eligibility for therapeutic options, it is important to analyze BrM tissues to obtain accurate biomarker information for appropriate treatment selection (58, 93). Importantly, most patients (63.6%) with biomarker discordance between the primary neoplasm and BrM also show discordance between extracranial metastases and BrM, with the primary and extracranial neoplasms typically being concordant (91, 92). Thus, different treatment options may have therapeutic activity in BrM that can currently only be identified by profiling BrM. For instance, anti-HER2 therapy (e.g., trastuzumab, pertuzumab, or lapatinib) can be used for HER2 amplification, which are frequently increased in BrM compared to primaries and extracranial metastases. Recently, newer HER2-targeted agents have shown an ability to reach BrM and generate intracranial responses (61). Excitingly, a recent exploratory analysis of 291 patients with BrM who were included in the HER2CLIMB randomized controlled trial demonstrated that the addition of tucatinib to trastuzumab and capecitabine doubled the intracranial response rate, highlighting a regimen that may be especially effective against HER2-positive BrM (14). Similarly, endocrine therapy (e.g., tamoxifen or aromatase inhibitors) can be applied to tumors with positive HR status (94, 95). While treatment options for TNBC BrM have historically been limited to chemotherapy, there is emerging evidence of effectiveness of androgen receptor (AR)-targeted therapies (e.g., bicalutamide or enzalutamide) in TNBC (62, 63).

Deletion of phosphatase and tensin homologue (*PTEN*) on chromosome 10 has been found in a significant portion of BCBrM (96). Furthermore, significantly decreased PTEN mRNA and protein expression has been observed in BCBrM compared to primary tumors (71, 97). Loss of PTEN may be a critical factor for BrM development, a possibility that is supported by research suggesting that the loss of PTEN is often exhibited in intracranial malignancies but less commonly in extracranial sites (64–66). Downregulation of PTEN expression has not been observed in bone metastases, suggesting that PTEN dysfunction may be uniquely supportive to metastatic growth in the brain microenvironment (97–99). PTEN antagonizes the phosphatidylinositol 3-kinase (PI3K)/AKT/mTOR pathway, with loss of PTEN resulting in aberrant activation of the pathway and enhanced tumor cell proliferation (100). Identifying PTEN loss in BrM opens the door to potential therapeutic modalities, including PI3K inhibition (7). It has also been suggested that loss of PTEN sensitizes malignant cells to polyadenosine diphosphate ribose polymerase (PARP) inhibition (67). Importantly, BrM profiling could potentially identify resistance mechanisms for PARP inhibitors to help rationally guide the selection of the next line of therapy. Additional therapeutic options targeting loss of PTEN in BrM require further investigation (68).

CDK4/6 inhibitors, including abemaciclib, palbociclib, and ribociclib, are another major class of treatment for BC metastases (7). Abemaciclib is the most brain permeable of the class and has been tested in a recent clinical trial of patients with HR-positive, HER2-negative BrM with promising results (101). Palbociclib has also demonstrated intracranial efficacy in patients with CDK pathway alterations and BrM in a basket trial, including in patients with BrM from breast cancer (102). However, clinical studies have linked homozygous retinoblastoma protein 1 (RB1) loss to resistance to CDK4/6 inhibitors (24, 73, 74). Homozygous RB1 loss has been observed more frequently in metastatic BC, especially BCBrM, as compared to primary tumor sites (24). RB1 mutations are linked to chromosomal rearrangements that subsequently disrupt genes that inhibit tumor growth and progression. Thus, molecular profiling of BCBrM may present additional treatment options, or may indicate potential resistance to additional options, for these patients.

### Melanoma

*BRAF* is a gene that encodes the B-Raf protein, which is a serine–threonine kinase. Activating mutations in BRAF, the majority of which are *BRAF^V600E^*, occur in approximately half of cutaneous melanomas (103). Previous studies have reported that up to 7% of *BRAF* mutations found in BrM are not found in primary melanoma sites (76, 77). Highly selective BRAF and MEK inhibitors (e.g., vemurafenib and dabrafenib) are now approved and demonstrate clinically meaningful activity in the brain (78). These results indicate that biopsies of BrM for subsequent BRAF analysis should be considered in select patients to guide treatment decisions.

### Immune Checkpoint Blockade

The treatment of patients with a variety of solid tumors has benefitted from immune checkpoint blockade (ICB). While patients with intracranial metastases were historically excluded from systemic and immunotherapy trials, intracranial responses are increasingly observed following ICB, prompting newer interest in harnessing immunotherapy for these patients. In particular, agents targeting the programmed cell death-1 (PD-1)/programmed cell death ligand-1 (PD-L1) axis, as well as cytotoxic T-lymphocyte-associated antigen 4 (CTLA-4), have been used clinically now across BrM from a number of primary disease indications and have been approved for use in melanoma and NSCLC (104, 105). Most famously, perhaps, dual checkpoint inhibitor therapy with ipilimumab and nivolumab demonstrated intracranial response rates of 52% in selected asymptomatic patients with active melanoma BrM (106). Overall survival (OS) in this study was 81.5% at 12 months, and median survival had not been reached at 30 months (106). Meanwhile, an early combined analysis of both lung and melanoma BrM patients from a further phase II study illustrated intracranial response rates to pembrolizumab (anti-PD-1) monotherapy of 33% and 22%, respectively, with nearly identical extracranial response rates (107). This may shift the indication for ICB to up front rather than salvage therapy, as a number of these studies were conducted in patients receiving no prior therapy for their intracranial disease, and high concordances between intracranial and extracranial disease were typical.

Despite some notable successes, optimal biomarkers to guide therapeutic decision-making are lacking. Previous studies have reported that up to 50% of PD-1 expression that was found in BrM was not found in the primary melanoma site (108). This has prompted the search for additional predictive biomarkers for ICB, including tumor mutational burden (TMB). TMB, the total number of non-synonymous mutations in the coding regions of genes, has recently emerged as a potential biomarker to select patients for immunotherapy. The FDA granted accelerated approval to pembrolizumab (KEYTRUDA, Merck & Co., Inc.) for the treatment of adult and pediatric patients with unresectable or metastatic TMB-high (TMB-H) solid tumors that have progressed following prior treatment and who have no satisfactory alternative treatment options (109). Metastatic tumors have increased TMB at recurrence, and BrM is found to have the highest level of TMB among metastatic sites (110, 111). Given emerging evidence of response to ICB in intracranial tumors (112–114), specifically evaluating TMB as a predictive biomarker is a priority that will require increased molecular profiling or BrM.

### Radiation Therapy Considerations

Radiation therapy has long been applied for BrM in a fashion that is largely agnostic to tumor histology. However, emerging evidence suggests that the genetic configuration of BrM could dramatically impact its response to radiation therapy. For instance, a recent pan-cancer analysis found that tumors containing pathologic genetic alterations in the apical DNA-damage response gene *Ataxia telangiectasia mutated (ATM)* have dramatically improved local control after radiation therapy compared to control tumors (incidence of irradiated tumor control 13% vs. 28% at 2 years) (115). This link between *ATM* pathogenic variants and radiosensitivity seems to extend to primary brain tumors (116). Thus, the mutational status of genes such as *ATM* may be one of several factors that, in the setting of a multidisciplinary BrM tumor board, could guide whether to approach a BrM with primary SRS, with surgery, or to reimage the brain following a trial of systemic therapy. Given the discordance between BrM and primary tumor genotype (24), sampling of the BrM *ATM* genotype would be expected to provide the most robust biomarker for radiosensitivity. Further validation of this finding and investigation of other genetic biomarkers that may be linked to radiosensitivity are warranted in the BrM setting.

## Identification of Ineffective Treatment Strategies

Molecular profiling of BrM can help indicate whether certain targeted therapies are likely ineffective in this setting. First, resistance to molecularly targeted therapies can occur over the course of treatment and render therapies ineffective against late-stage disease, including BrM. Drug resistance can develop through multiple mechanisms, including but not limited to restoration/reactivation of downstream targets, activation of alternative signaling pathways, and mutations in the binding site of a targeted protein that alter binding of the drug (117). This therapeutic resistance may develop after initial treatments of the primary neoplasm and other metastatic sites. As a result, treatment for BrM based on tissue samples from the primary tumor or other metastatic sites alone may misinform clinical decision-making. Second, actionable targets that were once present in the primary and/or extracranial tumors may be lost in the BrM. Thus, continued treatment with the original matched targeted therapy would be ineffective in the BrM and subject the patient to unnecessary side effects and costs. In this section, we discuss mechanisms of drug resistance and loss of biomarkers in BrM from NSCLC, breast cancer, and melanoma and discuss how knowledge of BrM biomarkers can guide therapy away from ineffective therapies.

### Non-Small Cell Lung Cancer

Although most NSCLC harboring an EGFR mutation are initially responsive to treatment with first-generation TKIs, the majority of patients develop drug resistance within 1–2 years (30). Approximately 60% of acquired resistance to early-generation TKIs is due to the acquiring of the EGFR T790M mutation (118). Tumors may also acquire resistance through activation of signaling molecules downstream of EGFR. Indeed, MET (N-methyl-N′-nitroso-guanidine human osteosarcoma transforming gene), a receptor tyrosine kinase that is considered an oncogenic driver in NSCLC (119–121), is suggested to be closely linked to the EGFR pathway (46–49) and its resistance to inhibitors (42, 50), and has been observed to have a higher rate of mutation in BrM versus primary NSCLCs (69, 70). Providers treating patients who progress after an EGFR TKI should consider molecular analyses of BrM tissue to confirm whether continued treatment with an EGFR inhibitor, or switching to a different TKI like the T790M mutant-specific, brain-penetrant inhibitor osimertinib (30, 31), will be effective this setting.

### Breast Cancer

As discussed above, activation of the PI3K/AKT/mTOR pathway, such as through loss of PTEN, has been suggested to play a role in the mechanisms underlying poor responses to anti-HER2 therapy in BC metastases (122, 123) and has been found to be altered in more than half of BCBrM (122, 124). However, targeting a single biomarker of the PI3K/AKT/mTOR pathway (e.g., PI3K, HER2, or HER3) is often ineffective (125, 126). Combination therapies aimed against multiple molecular targets (e.g., HER3+PI3K or PI3K+mTOR) appear to be more efficacious against BCBrM than monotherapy in preclinical models (125, 126).

HR-positive BC has a lower frequency of metastasizing to the brain compared to other BC subtypes (127). However, in those patients that do develop BrM, their disease has frequently become resistant to hormone therapy at this late stage of the disease through acquisition of HR mutations (7). Furthermore, BCBrM also frequently demonstrates loss of ER and PR. Indeed, a recent analysis showed that 14.8% and 22.4% of BCBrM had loss of ER and PR, respectively, contributing to the 22.8% of cases that had a subtype switch between primary or extracranial tumors and BrM (91, 92). Thus, hormone therapy may be ineffective in treating a significant portion of BrM given their frequent acquired resistance and/or loss of HR expression.

### Melanoma

Melanoma patients often develop treatment resistance within 1 year of receiving BRAF/MEK-targeted therapy. Agents which target the BRAF/MEK pathway have shown meaningful clinical activity in patients with melanoma BrM, although resistance has been observed to develop within a shorter period of time (78). Several mechanisms for treatment resistance have been suggested, including receptor tyrosine kinase upregulation (e.g., PDGFRß, IGF1R), acquisition of MEK alterations, and activation of the RAS/RAF/MAPK pathway (128).

A recent report comparing melanoma BrM to matched primary and extracranial melanoma tumors demonstrated biomarker discordance between BrM and extracranial sites in 5/8 patients, including loss of mutant NRAS (111). Of note, 2 patients with multiple BrM also showed some differences in potentially actionable alterations between the individual BrM. While overall concordance with extracranial metastases is felt to be high with respect to driver mutations, studies have revealed important molecular differences in melanoma BrM, such as increased activation of the PI3K/AKT pathway (129).

## Prognostic Information

Assessing the biomarker status of BrM is not only valuable for informing the treatment plan—both by adding new potential strategies and by ruling out ineffective ones—but can also provide prognostic information to improve patient and provider expectations for care. Prognostic information is particularly important to patients with BrM, as BrM symptoms are often associated with decreased functional status and severe reductions in quality of life.

### Non-Small Cell Lung Cancer

Studies suggest that BrM with driver mutations, including EGFR and ALK, were associated with longer overall survival when treated with surgery, radiosurgery, and non-surgical interventions (5, 130–138). Specifically, Zhou et al. report that BrM patients with common EGFR mutations treated with icotinib exhibited a prolonged PFS compared to those with uncommon EGFR mutations (32). There is a solid body of evidence suggesting that significant survival increases are associated with NSCLC BrM with EGFR mutations compared to those without EGFR mutations (80, 131, 137–139). A recent meta-analysis of 18 studies supports this conclusion and posits that this is likely due to treatment sensitivities of the metastatic lesions (131).

### Breast Cancer

A number of biomarkers hold prognostic value for BCBrM. Approximately 20%–25% of breast cancers have amplified HER2 status (140–142), which is associated with longer survival among BC patients with BrM (59). Clinical data suggest that increased survival associated with HER2 positivity is likely a reflection of treatment effects related to anti-HER2 therapy rather than a reflection of the HER2-associated biological composition of BrM (59).

As previously discussed, loss of PTEN may be a critical factor for BC metastases to develop in the brain parenchyma (64–66). Studies have shown that loss of PTEN was associated with decreased time to tumor recurrence in distant sites, including the brain, in BC metastases (71). Furthermore, loss of PTEN has been associated with worse overall survival in patients with TNBC (71, 72).

Hexokinase (HK2), which plays an essential role in glucose metabolism (143, 144), is overexpressed in BrM compared with primary breast tumors. Increased HK2 expression has been associated with decreased post-craniotomy survival in BC patients with BrM (75).

## Challenges and Future Considerations

Biomarker analyses of BrM offer potential clinical gains by identifying and/or eliminating candidate targeted therapies. Currently, clinicians do not always obtain biopsies or send resected BrM tissues for biomarker analyses, resulting in a missed opportunity to better inform patient care and potentially improve outcomes. Clinicians may also be daunted by the variety and complexity of biomarker testing options or not be aware of recent work in the genetics of BrM demonstrating biomarker discordance and sometimes unique genetic profile in these metastases. Furthermore, the application of targeted therapies to treatment of BrM is currently limited to those which can penetrate the blood–tumor/blood–brain barrier, providing an additional layer of complexity in screening potential therapeutic modalities. Providing clinicians access to biomarker testing, clearly summarized and annotated results, and to molecular tumor boards may help them to better appreciate the value and interpret results of biomarker profiling in BrM.

There are at least two potential reasons why clinicians may not seek to test BrM tissues for biomarkers despite the potential utility of this information. First, clinicians may not realize that biomarker analyses from BrM resections or biopsies can provide valuable information that is different from that obtained from the primary tumor or extracranial metastasis sites. Even when clinicians attempt to analyze BrM tissue for biomarkers, a large and growing number of complex testing options can present practical difficulties, particularly in resource-limited settings (145). Whole-transcriptome sequencing (WTS) and whole-exome sequencing (WES) platforms that are currently used for research purposes have recently become standard of care at many institutions and commercial providers. Pan-cancer whole-genome analyses of metastases have revealed therapy-associated mutations that contribute to drug resistance in individual patients (146–148). However, such analyses can be complex to interpret and utilize (149). Furthermore, practical considerations, such as which genetic testing platforms are FDA approved, and whether genetic tests are covered by insurance, can make it difficult to recommend additional genomic profiling in BrM. The growing number of testing options, and practical considerations for each, makes it increasingly difficult for clinicians to order and select the most appropriate biomarker analyses. In the future, development of targeted panels for types of primary tumors that metastasize to the brain could be considered to augment accessibility of BrM biomarker analyses for clinicians.

The optimal use of targeted BrM therapies depends largely on the expertise of clinicians (150), many of whom have limited experience considering the efficacy of targeted therapies in crossing the blood–brain barrier (151). As a result, management of BrM often requires a multidisciplinary approach (12), with molecular tumor boards being a vital venue for discussion of treatment plans with input from multiple specialties (152). Access to molecular tumor boards would likely improve and increase the application of genomically guided cancer care for patients with BrM, including targeted clinical trial enrollment. Data suggest that less than half of all hospitals and only 5% of non-academic hospitals have access to molecular tumor boards (149). Clinicians at hospitals treating patients with BrM may face logistical obstacles in accessing molecular tumor boards, such as long distances to in-person meetings, low local patient volume, and limited personnel, although the recent global shift toward increasing comfort with web-based conferencing may serve to accelerate adoption of online multidisciplinary tumor boards. Organizing molecular tumor boards across multiple hospitals or hospital systems to provide clinicians access to relevant expertise is a logical and critical step forward in advancing use of molecular tumor boards across sites (149).

## Conclusion

Targeted therapeutic strategies and prognostic stratifications for treatment of patients with BrM are increasingly common. Despite the fact that discordance often exists between BrM and both primary tumors and distant extracranial metastases, molecular profiling of resected BrM is not currently routine, and biopsies for the purpose of biomarker evaluation are rare. Biomarker information from BrM can identify new mutations with viable targeted therapies, eliminate agents from consideration when resistance or loss of actionable biomarkers has developed in the BrM, and improve prognostication. Clinicians may be initially dismayed by the variety and complexity of biomarker testing options, but this challenge can be overcome by (virtual) molecular-tumor boards to guide decision-making and advance personalized oncology care for patients with BrM.

## Author Contributions

Investigation: ES. Resources: KW, CRG. Data curation: ES, AV. Writing: ES, AV, MP, KB, RV, CB, BP, ZR, AS, JC, CA, PF, CRG, KW. Visualization: ES. Supervision: CRG, KW. Project administration: CRG, KW. All authors contributed to the article and approved the submitted version.

## Funding

CRG received grants from the Robert Wood Johnson Harold Amos Medical Faculty Development Program, Federal Food and Drug Administration, and the NIH/NINDS K12 NRCDP Physician Scientist Award. CA is a Translating Duke Health Scholar of Duke University, Durham, NC. ZR received a Pediatric Brain Tumor Foundation Early Career Development Award, a Defeat DIPG SoSo Strong Foundation ChadThough Foundation New Investigator Award, a St. Baldrick’s Foundation Fellowship, and developmental funds from Cancer Center Support Grant P30CA014236.

## Conflict of Interest

CA receives research funding from PUMA, Lilly, Merck, Seattle, Genetics, Nektar, Tesaro, G1-Therapeutics, ZION, Novartis, and Pfizer; a compensated consultant role from Genentech (1/2019–), Eisai (1/2019–), IPSEN (2/2019–), Seattle Genetics (11/15/2019–11/15/2020), Astra Zeneca (3/2020–6/2020), Novartis (5/2020–5/2022), Immunomedics (10/1/2020–9/22/2021), Elucida (9/2020), and Athenex (2/2021–2/2023); and royalties from UpToDate and Jones and Bartlett. AS receives research funding (paid to institution) from Bristol Myers Squibb, Immunocore and Merck and a compensated consultant role from Novartis, Pfizer, Iovance, and Regeneron. ZR receives royalties for intellectual property related to cancer molecular diagnostic testing that has been licensed to Genetron Health and is managed by Duke University. JC receives research funding (as PI) from Bristol-Myers Squibb, Genentech, Spectrum, Adaptimmune, Medpacto, Bayer, AbbVie, Moderna, GlaxoSmithKline, Array, AstraZeneca, Grid Therapeutics, and CBMG; speaker at Merck and AstraZeneca; adviser at AstraZeneca (10/2018, 10/26/19), Guardant (12/18/18), Merck (3/8/19), Pfizer (1/10/20), NGM Biopharmaceuticals (1/20/20), Spectrum (9/18/20), Genentech (11/1/20); travel from Merck, AstraZeneca, Pfizer, and NGM Bio; Board of Directors at Lung Cancer Initiative of North Carolina (uncompensated); and DMSC at G1 Therapeutics.

The remaining authors declare that the research was conducted in the absence of any commercial or financial relationships that could be construed as a potential conflict of interest.

## Publisher’s Note

All claims expressed in this article are solely those of the authors and do not necessarily represent those of their affiliated organizations, or those of the publisher, the editors and the reviewers. Any product that may be evaluated in this article, or claim that may be made by its manufacturer, is not guaranteed or endorsed by the publisher.
